# Quality of Life among General Populations of Different Countries in the Past 10 Years, with a Focus on Human Development Index: A Systematic Review and Meta-analysis

**Published:** 2017-01

**Authors:** Fatemeh KOOHI, Saharnaz NEDJAT, Mehdi YASERI, Zahra CHERAGHI

**Affiliations:** 1. Dept. of Epidemiology and Biostatistics, School of Public Health, Tehran University of Medical Sciences, Tehran, Iran; 2. Knowledge Utilization Research Center, Tehran University of Medical Sciences, Tehran, Iran; 3. Dept. of Epidemiology, School of Public Health, Hamadan University of Medical Sciences, Hamadan, Iran

**Keywords:** Quality of life (QOL), Human development index (HDI), General population, Meta-analysis

## Abstract

**Background:**

The current study was conducted to estimate the integrated mean of Quality Of Life (QOL) of the general population of different countries around the world and to compare them on the grounds of the Human Development Index (HDI).

**Methods:**

Well-known international databases such as Medline, Scopus, Science Direct, Google Scholar & Google, and domestic databases including SID, IranMedex, Irandoc & Magiran were searched in 2015. The data were analyzed with the Random Effect Model, using Stata 11 software.

**Results:**

Ninety-seven studies were selected for the final analysis. The overall QOL mean in the very high HDI subgroup was 74.26 (CI=72.40–76.12), which was the highest value. The lowest mean score was observed in the psychological domain (M=67.37; CI=66.23–68.52). In the high HDI subgroup, the highest mean was observed in the social relationships domain (M=64.16; CI=61.99–66.34), and the lowest mean was observed in the environment domain (M=58.76; CI=56.50–61.03). In the medium HDI subgroup, the highest mean was calculated for the overall QOL score (M=62.62; CI=56.35–68.92), and the lowest mean was estimated for the environment domain (M=56.98; CI=53.54–60.43). The highest mean in the low HDI subgroup was observed in the physical health domain (M=68.17; CI=67.43–70.52), and the lowest mean was calculated for the environment domain (M=53.14; CI=51.57–54.72). There was considerable heterogeneity in all the subgroups and domains; the values reported here are the weighted means of QOL for different countries.

**Conclusion:**

Overall, the highest means of various QOL domains were observed in the very high HDI subgroup.

## Introduction

Nowadays, the measurement and assessment of quality of life among the general population have become one of the main activities of public health research. The results of these studies are utilized in resource allocation decision-making relevant to health promotion and well-being (1). Generally, Quality of Life has no definite and universal definition, although people instinctively understand its meaning, but the concept is not the same among them (2).

Most experts consider QOL as a subjective and dynamic concept. Subjective means the individual’s own opinions must be obtained, and dynamic means changes with time; therefore, it must be measured for a period. On the other hand, subjective measurement is necessary, but not sufficient, and that each of the QOL domains should have the ability to be measured both subjectively and objectively (2).

According to the definition of World Health Organization (WHO), quality of life is people’s concept of their positions in life from the perspective of the culture and evaluative system in which they live, and the goals, expectations, standards and priorities that they have. The factors that affect individuals’ concepts of QOL are physical health, psychological status, level of independence, social relationships, personal beliefs and environmental characteristics (3).

QOL somewhat describes the status of the people living in a country or region, and is nowadays considered an acceptable theoretical framework for examining the living conditions of different societies. In addition to economic issues, QOL affects the statuses of a society’s individuals, taking into account exogenous factors such as infrastructures, social organizations, social relationships, environment etc. (4). Information on QOL of the general population of a given country can provide basic data for assessing interventions (5).

The WHOQOL-BREF questionnaire is a multicultural QOL assessment tool that consists of four broad domains, physical health, psychological health, social relationships and environmental health, and another two questions that evaluate the overall QOL and general health status (6–8). This questionnaire has been translated and validated into over 40 languages around the world (7).

The human development index (HDI) was introduced in 1990 as a new index for measuring development in different communities. This index is based on the basic idea that the prerequisite of achieving a better life, in addition to having a high income, is the flourishing and development of human talents and capacities (9). The HDI is a composite index for assessing the success achieved by a given country in three key dimensions of human development: a long and healthy life, access to knowledge, and a decent standard of living (10).

HDI is the geometric mean of normalized indices for each of the three dimensions, calculated using the following:

- Life expectancy,
- Expected years of schooling (of children), mean years of schooling (the average numbers of years of education received by the population aged ≥ 25 yr in a country –without the years repeated);
- The gross national income per capita (GNI) (which is the average income of the citizens of a country -in dollars- and is calculated by dividing the entire income of a country –in dollars- by the population of that country) (11).

Each year, the United Nations Development Program (UNDP) publishes an annual report in which the HDI of all the countries have been calculated and ranked in comparison to the others in that given year (11).

The calculated HDI is a number between zero and one. Through this index, the UNDP classifies the countries around the world into four groups: countries with a very high HDI (HDI≥0.8); countries with a high HDI (0.8>HDI≥0.7); countries with a medium HDI (0.7>HDI≥5.5); and countries with a low HDI (HDI≤5.5) (11 ,12).

There is no published study on the association between HDI and QOL among general population of different countries. The current study was conducted to explore the mentioned association for hypothesis generation

## Materials and Methods

### Source of data

This study was a systematic review conducted on QOL of general populations of different countries around the world in the past 10 years, with an emphasis on HDI. To collect the required data, articles and thesis, the following keywords were used: quality of life, WHO’s quality of life instrument, WHOQOL-BREF, and a combination of these keywords. International databases such as Medline, Scopus, Science Direct, Pro-Quest, Google Scholar & Google, and domestic databases including SID, IranMedex, Irandoc & Magiran were searched. In order to find additional relevant articles, we scanned the reference lists of all retrieved studies. In addition, we contacted authors of retrieved studies for additional unpublished studies.

### Inclusion criteria

**Fig. 1:**
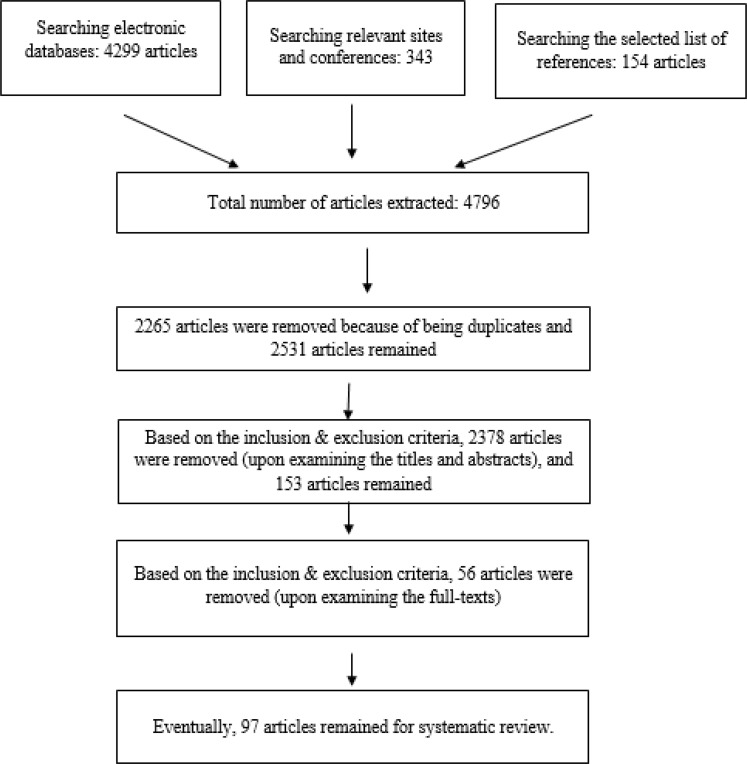
Flowchart depicting the stages of entry of studies into the systematic review & meta-analysis

All the articles and thesis published between the years 2004 & 2014 applied the WHOQOL-BREF questionnaire to assess QOL among the general population - irrespective of language of publication and we have 24 non-English publications. Studies not conducted on general populations, on the latter but not reported the mean QOL, insufficient data, and whose inaccessible full texts were excluded. Thus, 4796 articles relevant to QOL were found. Among these, 2265 articles were removed because of being duplicates, and 2378 articles because of their irrelevancy (upon examining the topics and abstracts). A hundred and fifty-three articles remained, out of 56 removed for having inadequate data (upon reading the full-texts). Eventually, 97 articles of appropriate quality were systematically reviewed (Fig. 1).

### The WHOQOL-BREF questionnaire

The WHOQOL-BREF questionnaire is a multi-lingual, multicultural instrument designed to evaluate QOL. It consists of 26 items extracted from the WHOQOL-100 questionnaire. It has four domains, physical health (7 questions); psychological health (6 questions); social relationships (3 questions); environmental health (8 questions); and two more questions that assess the overall QOL and health status (6–8).

In this questionnaire, the physical health domain covers items such as activities of daily living, fatigue & energy, pain & sorrow, working capacity, sleep & rest, motor strength, dependence on drugs, and medical goals. The psychological domain covers the individual’s image of his/her bodily appearance, negative feelings, positive feelings, self-confidence, thoughts, learning, memory, ability to concentrate, religion, beliefs, and sexual activity. The environment domain examines items such as financial resources, freedom & physical safety, social & health care, the physical environment of the living place, existing opportunities for gaining new skills & information, leisure opportunities, physical environment (noise pollution, air pollution, etc.) and transport (5).

After performing the necessary calculations for each domain, scores ranging from 4 – 20 result; 20 indicating the best and 4 indicating the worst status of QOL in the respective domain. These scores can be converted into scores within a range of 0–100. This questionnaire has been translated and validated into over 40 languages worldwide, and is still in progress (7).

This questionnaire has been translated and standardized in Iran, too; upon test –retest after two weeks an intraclass correlation of 0.75–0.84 was obtained for all the four domains, which indicates its reliability. Furthermore, Cronbach’s alpha and indices relevant to construct validity indicate the desirable validity of the tool in the Iranian population (13).

### Assessing the quality of studies

The quality of studies was assessed with the STROBE checklist, which is a standard checklist. Using this checklist, we assessed the reporting of the following items:

1. Type;
2. time and setting
3. the demographic characteristics of the individuals under study;
4. the inclusion and exclusion criteria;
5. method of measurement of variables;
6. the statistical methods applied;
7. standard deviations and confidence intervals of the estimates.

Upon examining the aforementioned items, the studies were classified into three groups: slightly biased and of high quality (100% adherence to the STROBE criteria), moderately biased and of average quality (adherence to at least 85% of the STROBE criteria), highly biased and of low quality (adherence to at least 70% of the STROBE criteria).

### Data extraction

The data of the articles included in the systematic review were extracted using a previously developed checklist. Most of the studies’ data were extracted based on gender. This checklist included the study title, publication date, study setting, first author’s name, sample size (based on gender), the overall mean & standard deviation (SD) of QOL, mean & SD of the physical health domain, mean & SD of the psychological health domain, mean & SD of the social relationships domain, and, mean & SD of the environmental health domain.

### Statistical analysis

Since the QOL domains’ means & standard errors had been reported with different scorings in different studies, they were transformed to the 0–100 scale using the transformation formula before the meta-analysis began. We did not examine publication bias, because our objectives for this systematic review were descriptive in the descriptive studies the publication of the articles is not affected by the low or high prevalence (14).

We examined the heterogeneity of the studies with the Chi^2^ test and the I^2^ & T^2^ indices. Bearing in mind the considerable heterogeneity between studies, the Random Effect Model has been used with caution in combining the study results.

Here, we needed an integrated mean to compare the HDI subgroups, thus, the meta-analysis was conducted through a random approach. However, the latter indicator was only considered as a weighted mean of the domain scores, and not in estimating QOL in the countries of those subgroups (15, 16). In this study, we used the data presented in the UNDP HDI Report of 2014. Accordingly, the countries included in the report have been classified into four subgroups: very high HDI (HDI≥0.8); high HDI (0.8>HDI≥0.7); medium HDI (0.7>HDI≥5.5); and low HDI (HDI≤5.5))11). The data were analyzed with Stata 11 software. Moreover, to prove the significance of the association between the mean QOL scores and HDI, the *P*-value of the Wald test was calculated using meta-regression in which the overall QOL mean and its domains were entered as dependent variables, and the HDI was entered as the independent variable.

## Results

The number of individuals participating in the studies was 100214, with a mean age of 46.59±3.68 yr.

Based on heterogeneity test results, the Chi^2^ test was significant for all the subgroups. The I^2^ index also indicated >90% heterogeneity in all the subgroups. The QOL weighted mean in each HDI subgroup was estimated through the random effect model (Table 1).

**Table 1:** Estimating the integrated mean of QOL and its domains based on HDI, using the random effect model

** Quality of life **	** Subgruop **	** Number of article **	** Pooled mean (95% CI) **	** Chi- square **	*** P * -value **	** I-square **	** Tue-square **
Overall quality of life	Very high human development index	24	74.26(72.40–76.12)	461.42	<0.001	95.00	19.16
	High human development index	43	64.10(61.95–66.24)	5021.00	<0.001	99.2	49.95
	Medium human development index	9	62.62(56.35–68.92)	1229.14	<0.001	99.3	91.85
	* Low human development index	2	65.57(63.13–68.02)	1.38	0.241	27.4	0.86
Physical domain	Very high human development index	66	70.06(68.18–71.95)	12373.73	<0.001	99.5	59.71
	High human development index	81	63.45(61.22–65.67)	23358.84	<0.001	99.7	102.70
	Medium human development index	15	62.26(57.15–67.37)	2320.43	<0.001	99.4	100.61
	* Low human development index	2	68.17(67.43–70.52)	0.90	0.343	0.00	111.73
Psychological domain	Very high human development index	66	67.37(66.23–68.57)	5763.62	<0.001	98.9	21.53
	High human development index	81	62.73(60.89–64.57)	16405.74	<0.001	99.5	69.42
	Medium human development index	15	58.25(52.58–63.93)	2882.96	<0.001	99.5	124.22
	* Low human development index	2	66.45(52.58–63.93)	0.00	0.949	0.00	0.00
Social domain	Very high human development index	66	69.88(68.70–71.06)	4029.95	<0.001	98.4	22.60
	High human development index	81	64.16(61.99–66.34)	18649.42	<0.001	99.6	98.10
	Medium human development index	15	62.06(56.95–67.17)	2167.95	<0.001	99.4	100.15
	* Low human development index	2	67.57(65.78–69.37)	0.11	0.744	0.00	0.00
Environmental domain	Very high human development index	64	70.05(68.19–71.91)	10139.77	<0.001	99.4	56.65
	High human development index	81	58.76(56.50–61.03)	24186.44	<0.001	99.7	105.80
	Medium human development index	15	56.98(53.54–60.43)	1174.94	<0.001	98.8	45.28
	* Low human development index	2	53.14(51.57–54.72)	0.19	0.664	0.00	0.00

* Because of the low number of articles in this subgroup, the I^2^ and T^2^ were zero

**Fig. 2:**
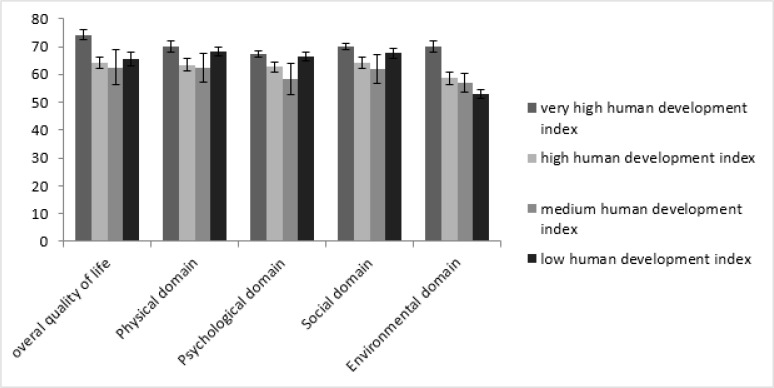
Histogram illustrating the integrated mean of QOL domains based on HDI

**Fig. 3:**
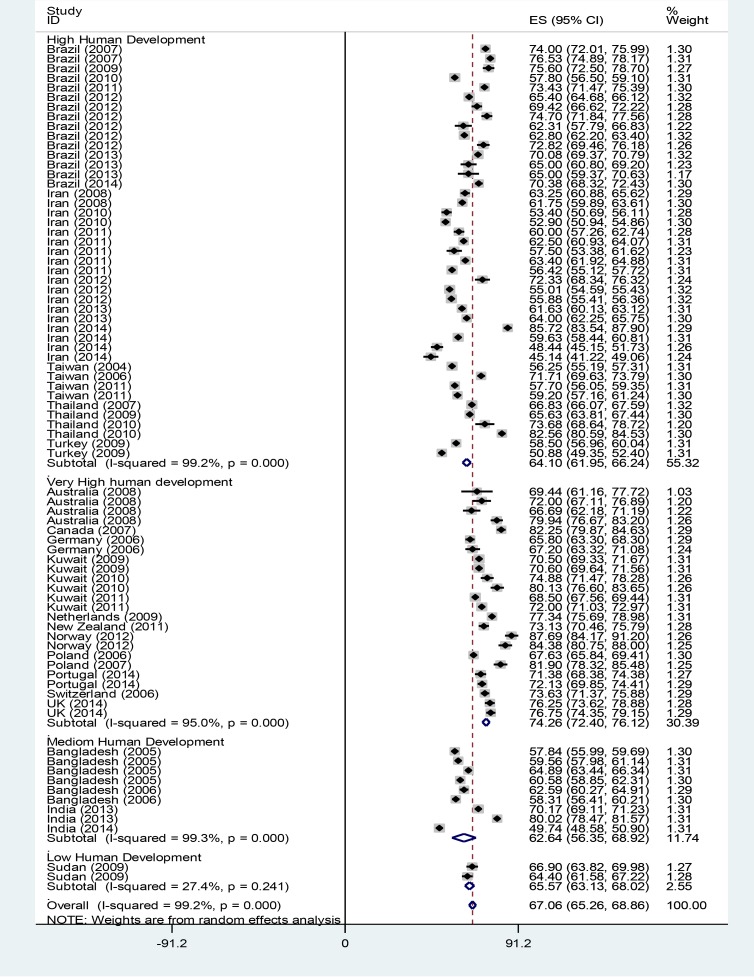
Estimating the overall integrated mean of QOL based on HDI in the Random Effect Model Each of the dashes represents the 95% confidence interval. The diamond sign represents the integrated mean of QOL and confidence interval of each subgroup

The QOL weighted mean in each sex subgroup was estimated through the random effect model and is shown in Table 2. Fig 2 shows the histogram illustrating the integrated mean of QOL domains based on HDI. Fig 3 shows the forest plot related to Estimating the overall integrated mean of QOL based on HDI in the random effect model.

**Table 2:** Estimating the integrated mean of QOL and its domains based on sex, using the random effect model

** Quality of life **	** Subgroups **	** Number of articles **	** Pooled mean (95% CI) **	** Chi- square **	*** P * -value **	** I-square **	** Tue- square **
Overall quality of life	male	24	65.42 (62.41–68.42)	2181.96	<0.001	98.9	53.73
	female	26	65.66 (62.65–68.66)	3685.74	<0.001	99.3	59.67
	both	28	69.75 (65.91–73.60)	3162.03	<0.001	99.1	106.32
Physical domain	male	47	66.20 (63.01–69.38)	14122.83	<0.001	99.7	122.16
	female	52	64.81 (61.99–67.63)	15957.87	<0.001	99.7	106.29
	both	65	67.01 (64.32–69.69)	22307.11	<0.001	99.7	120.80
Psychological domain	male	47	65.11 (63.36–66.86)	4493.47	<0.001	99.0	35.71
	female	52	62.35 (60.81–63.89)	6128.94	<0.001	99.2	30.36
	both	65	65.15 (62.69–67.61)	18863.82	<0.001	99.7	101.23
Social domain	male	47	65.92 (63.34–68.50)	7375.63	<0.001	98.4	78.85
	female	52	66.68 (64.54–68.83)	7834.34	<0.001	99.3	60.06
	both	65	66.33 (63.39–69.26)	22459.17	<0.001	99.7	143.83
Environmental domain	male	46	61.95 (58.41–65.51)	15164.74	<0.001	99.7	148.96
	female	51	61.73 (58.60–64.85)	17901.04	<0.001	99.7	128.20
	both	65	64.75 (62.10–67.40)	21765.11	<0.001	98.7	117.59

Based on the results of quality assessment, the studies fell into the following categories: slightly biased and of high quality (53 cases; 54.64%), moderately biased and of average quality (34 cases; 35.05%), highly biased and of low quality (10 cases; 10.31%).

Based on the results of the subgroups’ analysis in Table 1, the overall QOL score had the highest integrated mean in the very high HDI subgroup (M=74.26). The means of the physical, environment and social domains ensued, and the lowest mean score was observed in the psychological domain (M=67.37).

In the high HDI subgroup, the highest mean was observed in the social relationships domain (M=64.16). The next highest means were observed in the overall QOL, physical and psychological domains. The lowest mean was observed in the environment domain (M=58.76).

In the medium HDI subgroup, the highest mean was calculated for the overall QOL score (M=62.62), followed by the means of the physical, social and psychological domains. The lowest mean was estimated for the environment domain (M=56.98).

The highest mean in the low HDI subgroup was observed in the physical health domain (M=68.17), followed by the means of the psychological, social and overall QOL. The lowest mean was calculated for the environment domain (M=53.14).

The highest mean for the overall QOL -based on the HDI- was observed in the very high HDI subgroup (M=74.26), followed by the low and high HDI subgroups. The medium HDI subgroup had the lowest overall QOL mean (M=62.62). Hence, regardless of the low HDI in the other subgroups, a positive association was observed between the HDI and the overall QOL, such that an increase in the HDI was associated with an increase in the overall QOL score (*P*<0.001).

In the physical health domain, countries with very high HDI gained the highest mean for the physical health domain (M=70.06), and the countries with medium HDI garnered the lowest mean (M=62.26). In this domain too, apart from the low HDI subgroup, a direct association was observed between the physical health mean and HDI; an increase in HDI was associated with an increase in the physical health mean (*P*<0.001).

In the psychological domain, the highest QOL mean (M=67.37) was observed in countries with very high HDI and the lowest mean (M=58.25) was observed in the countries with medium HDI. In this domain too, apart from the low HDI subgroup, a direct association was observed between the psychological domain’s mean and HDI; an increase in HDI was associated with an increase in the mean (*P*<0.001).

In the social relationships domain, countries with very high HDI gained the highest QOL mean (M=69.88), followed by countries with low and high HDI. The lowest mean (M=62.06) was observed in the medium HDI subgroup. In this domain too, apart from the low HDI subgroup, a direct association was observed between the social relationships’ mean and HDI; an increase in HDI was associated with an increase in the mean (*P*<0.001).

In the environment domain, unlike the other domains, a directly positive association was observed between the countries’ QOL and HDI. The very high HDI subgroup had the highest QOL mean in the environment domain (M=70.05), followed by the high HDI and medium HDI subgroups. The low HDI subgroup had the lowest environment mean (M=56.98) (*P*<0.001).

## Discussion

In the current study, despite the considerable heterogeneity, the random effect model was applied to combine the study results and to estimate the integrated mean for comparing the HDI subgroups. Here, we used the random effect model to estimate the integrated mean of QOL and its HDI-based domains, and observed the highest integrated means to be in the very high HDI subgroup. The overall QOL was the highest (M=74.26; CI=72.40–76.12) in the very high HDI subgroup, and the lowest integrated mean (M=67.37; CI=66.23–68.52) was calculated for the psychological domain in the high HDI subgroup.

Overall, our results indicate a positive association between QOL and HDI. The low HDI subgroup excluded, the mean scores directly increased with an increase in HDI in the remaining HDI subgroups in the physical health, psychological, social relationships and overall QOL domains. A direct positive association with the HDI subgroups was observed only in the environment domain. The high overall QOL mean observed in the low HDI subgroup may be attributed to the small number of articles in this subgroup, led to the insignificance of the heterogeneity test and resulted in the zero value of the I^2^ and T^2^ indices. Few studies have been conducted on the association between QOL and HDI (17). Totally, 11801 persons aged 12–97 yr from 23 countries studied, with the goal of investigating QOL, educational status and HDI, using the WHOQOL-BREF (17). Eventually, 9404 persons from 13 countries were investigated because of the completeness of their data. The results showed a good overall QOL mean; the social relationships and environment domains exhibited the highest and lowest means, respectively, findings that are consistent with ours. Furthermore, although the social QOL was good in all the countries and no difference was observed between two HDI groups, developing countries and those with lower HDI (the medium HDI subgroup) exhibited lower QOL in the physical, psychological and environment domains when compared to developed countries and those with higher HDI. These findings too, are consistent with our results (17).

Once again, using the WHOQOL-BREF, the association between human development and QOL in Brazil, on 182 individuals aged over 60 yr, from three different cities of the State of São Paulo (City **A**–with the highest HDI, City B, & City C–with the lowest HDI). The results of this study differed from ours, such that no significant difference was observed between the cities’ overall QOL mean scores. However, significant associations were observed between the social and environment domains of QOL and HDI. In line with our findings, the best QOL results of these two domains were found among the residents of cities **A** & **B**, which had the highest HDI, as opposed to City **C** –which had the lowest HDI (18).

Another study in Brazil, on 48 individuals from Belo Horizonte (with a high HDI=0.828) and 29 individuals from Montes Claros (with a medium HDI=0.691). The participants had experienced the stroke in the last three years of their lives. QOL was assessed with the SF-36 questionnaire. In spite of the differences in HDI, no significant difference was observed between the QOL of the cities, findings that do not conform to ours (19). One of the limitations of our study was the high and considerable heterogeneity observed among the studies, which made the combination of studies impossible even with the random effect model. Nonetheless, this method was applied in light of the goals of the study –which meant to utilize the results in health policy making. Thus, the random effect model was used to combine the studies and to compare them with each other to estimate the weighted QOL means in different subgroups (20). Another limitation was that these 97 studies were related to only 32 countries around the world; the data relevant to the QOL of other countries were not available. The small number of studies in the low HDI subgroup is another limitation. Nevertheless, bearing in mind the type of indicator under study, although the number is small, we could imagine similar countries to have similar conditions, and therefore, use the results to predict the status of other countries as well.

## Conclusion

We can present an overall estimate of QOL and its domains among the general populations around the world to prove beneficial in health planning. Moreover, we may compare the QOL of different HDI subgroups. HDI can be used as a predictor of QOL. Thus, efforts must be aimed at promoting the HDI determinants. In this respect, the level of health should be promoted to increase life expectancy, improve educational status, and raise monthly incomes in various countries.

## Ethical considerations

Ethical issues (Including plagiarism, informed consent, misconduct, data fabrication and/or falsification, double publication and/or submission, redundancy, etc.) have been completely observed by the authors.
